# Gut-Brain Axis Impact on Canine Anxiety Disorders: New Challenges for Behavioral Veterinary Medicine

**DOI:** 10.1155/2024/2856759

**Published:** 2024-01-23

**Authors:** Carina Sacoor, John D. Marugg, Nuno R. Lima, Nuno Empadinhas, Liliana Montezinho

**Affiliations:** ^1^Vasco da Gama Research Center (CIVG), Vasco da Gama University School (EUVG), 3020–210 Coimbra, Portugal; ^2^Department of Veterinary Sciences, School of Agrarian and Veterinary Sciences (ECAV), University of Trás-os-Montes e Alto Douro (UTAD), 5000–801 Vila Real, Portugal; ^3^Center for Neuroscience and Cell Biology (CNC), University of Coimbra, 3004–504 Coimbra, Portugal; ^4^Center for Innovative Biomedicine and Biotechnology (CIBB), University of Coimbra, 3004–504 Coimbra, Portugal; ^5^Animal and Veterinary Research Centre (CECAV), UTAD, and Associate Laboratory for Animal and Veterinary Science (AL4AnimalS), 5000–801 Vila Real, Portugal; ^6^Innovation in Health and Well-Being Research Unit (iHealth4Well-Being), Polytechnic Health Institute of North (IPSN-CESPU), 4585-116 Gandra, Portugal

## Abstract

Anxiety disorders in dogs are ever-growing and represent an important concern in the veterinary behavior field. These disorders are often disregarded in veterinary clinical practice, negatively impacting the animal's and owner's quality of life. Moreover, these anxiety disorders can potentially result in the abandonment or euthanasia of dogs. Growing evidence shows that the gut microbiota is a central player in the gut-brain axis. A variety of microorganisms inhabit the intestines of dogs, which are essential in maintaining intestinal homeostasis. These microbes can impact mental health through several mechanisms, including metabolic, neural, endocrine, and immune-mediated pathways. The disruption of a balanced composition of resident commensal communities, or dysbiosis, is implicated in several pathological conditions, including mental disorders such as anxiety. Studies carried out in rodent models and humans demonstrate that the intestinal microbiota can influence mental health through these mechanisms, including anxiety disorders. Furthermore, novel therapeutic strategies using prebiotics and probiotics have been shown to ameliorate anxiety-related symptoms. However, regarding the canine veterinary behavior field, there is still a lack of insightful research on this topic. In this review, we explore the few but relevant studies performed on canine anxiety disorders. We agree that innovative bacterial therapeutical approaches for canine anxiety disorders will become a promising field of investigation and certainly pave the way for new approaches to these behavioral conditions.

## 1. Introduction

In modern societies, dogs assume important roles, mainly due to their companionship [1], as they are considered family members [2]. Apart from dogs' intrinsic characteristics, their owners' lifestyles, socioeconomic conditions, work routines, and specific personality traits can potentiate canine behavioral problems. These include anxiety disorders, which represent a serious concern for animal welfare, and can further impact every other aspect of human society, including the economy, public health, and others. The combination of genetic factors, environment, and experiences in dogs' lives determines their behavioral development [1].

Anxiety is defined as a “preparatory response made in anticipation of threatening stimuli or scenarios” [3]. This adaptative response is the root cause of many canine behavior problems [3] and can result in a chronically stressful life for dogs [4]. An estimated 72.5% of dogs showed anxiety-like behaviors to a certain extent [4]. The Lincoln Canine Anxiety Scale has been validated as a reliable measurement to assess canine anxiety, which classifies this condition based on the severity of typical behaviors (Table 1) [3], which, from the most common to the least, include noise sensitivity (32%), fear (29%), excessive activity (15%), compulsive behavior (16%), aggression (14%), and separation-related behaviors (6%) [4].

Dogs with anxiety disorders are reported to be more vulnerable to other diseases and have shorter lifespans [4]. Additionally, these traits, particularly aggressiveness, might be of public health importance [5]. Anxiety disorders impact dogs' and owners' quality of life and affect their bond [6]. Plus, it is likely to result in dogs being abandoned or even euthanized [7]. This is a growing concern and requires further research [6]. To this end, the role of the gut microbiome in the pathophysiology of these behavioral disorders has recently started to be investigated.

Although research has addressed the influence of microbiota on anxiety disorders in humans and rodent models, there is a lack of insightful information on canine anxiety disorders. In this review, we intend to highlight recent data on the role of the microbiota in the gut-brain axis (GBA) and its impact on anxiety disorders in human and rodent models and, most importantly, to emphasize the exploratory yet relevant research applied to canine anxiety disorders. We also aim to highlight the positive outcomes that enhanced research in this area will provide for the future of behavioral veterinary medicine, the wellbeing of dogs, and the subsequent effect on society.

## 2. Materials and Methods

In this review, we provide a comprehensive overview of the gut-brain axis and its impact on anxiety disorders, summarizing the experimental and clinical evidence on the mechanisms involved and their influence on mental health. The revision was mostly based on scientific articles published between 2015 and 2022, as well as a few earlier works. The research was conducted on the PubMed and Google Scholar databases after removing duplicate references. The Medical Subject Headings (MeSH) terms “anxiety,” “gut-brain axis,” “hormones,” “immune cells,” “microbiota,” “neurotransmitters,” “intestinal permeability,” “zonulin,” prebiotics,” and “probiotics” were cross-referenced and used in the search platforms. Experimental or observational studies in humans, rodents, and dogs were identified and compared. Relevant clinical and experimental articles written in the English language have been included.

## 3. The Canine Microbiome and the Gut-Brain Axis

### 3.1. Microbiome

The term microbiome is defined as a “characteristic microbial community occupying a reasonable well-defined habitat which has distinct physiochemical properties” [8]. This term not only includes the microbiota, which are the microorganisms involved, but also encompasses the environmental conditions, microbial structural elements, and metabolites. The microbiota encompasses groups of bacteria, fungi, algae, archaea, and protists inhabiting the gastrointestinal tract [8]. This population of microorganisms is essential to support and promote the host's health [2, 9], as they participate in a large variety of physiological processes, including energy demand, metabolic reactions, immunological responses, and neurobehavioral development [10].

In healthy dogs, the gut microbiota is dominated by members of the phyla *Bacteroidetes*, *Fusobacteria*, and *Firmicutes* and, to a lesser extent, of the phyla *Actinobacteria* and *Proteobacteria* [2, 9, 11]. A high abundance of members of the genera *Fusobacterium*, *Prevotella*, and *Bacteroides* was detected in a metagenomic study that analyzed the feces of six wolves and 169 dogs from different breeds [9]. Several environmental factors, such as diet [9], body weight [2], age [12], and others, can influence the gut microbiota structure and composition. However, the impact of these factors is mild compared to the changes potentially induced by diseases [11].

Gut dysbiosis, defined as an altered composition of microbes [8], can lead to alterations in the microbial transcriptome, proteome, or metabolome [11]. Imbalances in the gut microbiota population are linked to pathological states, such as inflammation, obesity, metabolic alterations, and even mood disorders. Further, gut microbiota affects physiological, neuronal, and behavioral functions [13]. Therefore, homeostasis in this ecosystem is fundamental for maintaining the overall health condition [10].

### 3.2. The Gut-Brain Axis

**Figure 1:**
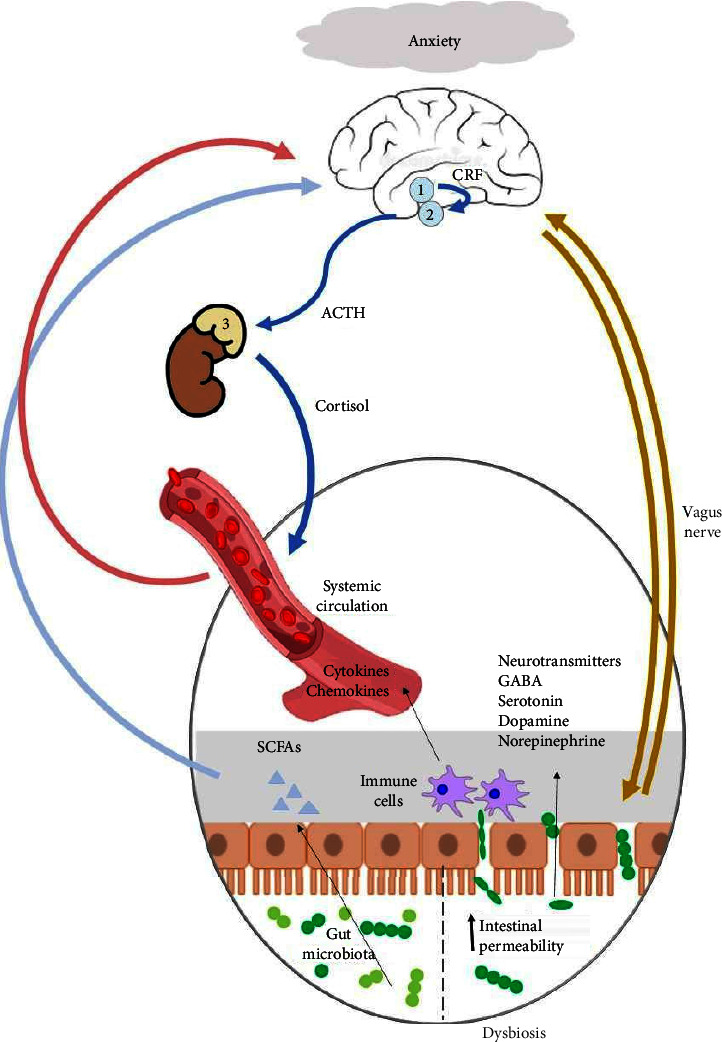
Mechanisms involved in the GBA. Anxiety disorders may lead to the hyperactivation of the HPA axis, causing the release of cortisol into the systemic circulation. In an inflammatory environment, characterized by gut dysbiosis and increased intestinal permeability, microbiota can translocate through the intestinal barrier. Moreover, immune cells may produce proinflammatory cytokines, while the gut microbiota itself may produce metabolites such as SCFAs and neurotransmitters that can directly impact mental health. Original illustration based on [15, 16] captioned as shown above: 1–hypothalamus, 2–pituitary gland, 3–adrenal gland, ACTH–adrenocorticotropic hormone, CRF–corticotropin–releasing factor, and SCFAs–short chain fatty acids.

The gut microbiome communicates bidirectionally with the host central nervous system through the GBA, a complex network capable of regulating cognitive functions and behavior through several different mechanisms, including neural, metabolic, endocrine, and immune-mediated signaling pathways [14]. The mechanisms involved in this regulation are summarized in Figure 1.

#### 3.2.1. Metabolic Pathways

The intestinal microbiome and the host have a complex symbiotic relationship [8]. Gut bacteria produce metabolites, such as short-chain fatty acids (SCFAs), from undigested dietary fibers that reach the colon, obtaining energy for their metabolism. SCFAs, including acetate, propionate, and butyrate, are essential for intestinal homeostasis [17], as they represent the primary energy sources for luminal colon cells [18]. In addition to the nourishment of intestinal epithelial cells, another important function is the maintenance of intestinal barrier functions [17]. Of particular interest, butyrate is reported to have a major role in supporting intestinal barrier integrity through the modulation of the expression of tight junction proteins, which greatly influence intestinal permeability [19]. SCFAs also possess anti-inflammatory and immunomodulatory effects [17], as they can modulate innate and adaptive immune responses [20]. Furthermore, SCFAs are also capable of crossing the blood-brain barrier (BBB), influencing endocrine responses [18, 21], and signaling the brain through vagus afferent fibers [18]. Propionate was found to protect the BBB from oxidative stress through nuclear factor erythroid 2-related factor 2 signaling and to inhibit pathways linked to microbial infections [21]. Supplementation with butyrate and other SCFAs restored brain function in rodents, with marked improvements in stress behaviors and no significant impact on microbial diversity [22, 23]. Diet and microbiota composition influence the production of these organic acids [19]. A decrease in microbiota diversity and reduced ratios of SCFA-producing bacteria was observed in subjects with generalized anxiety disorders, suggesting that lower SCFAs production could affect the intestinal barrier function [24]. Furthermore, SCFAs have been shown to attenuate cortisol response to acute stress, suggesting their potential influence on psychobiological processes [18].

#### 3.2.2. Neural Mechanisms

Communication through the GBA is ensured by the autonomic nervous system. Vagus nerve neurons can perceive microbiota metabolites at the mucosa level, such as SCFAs, through its afferent fibers, which are distributed in the intestinal wall. The gut information is transmitted to the central nervous system, and consequently, a response can be generated. Moreover, vagus nerve fibers integrate a cholinergic anti-inflammatory system, which can decrease peripheral inflammation and reduce intestinal permeability. Thus, stress stimuli might inhibit a vagal tone and affect gut microbiota composition and intestinal health [25].

The gut microbiota can interfere with neural mechanisms by regulating neurotransmitter levels, including serotonin (5-HT), dopamine, norepinephrine, and gamma-aminobutyric acid (GABA). Microbiota can produce and contribute to regulate their metabolic pathways in intestinal cells, thus influencing nervous system signaling and, ultimately, mental health [26].

5-HT, synthesized from L-tryptophan, is mainly produced in the gut's enterochromaffin cells and neurons from the enteric nervous system [27], playing a critical role in eating, arousal, sleep, cognition, social interactions [28], anxiety, and mood regulation [28–31]. Gut microbiota can directly or indirectly influence tryptophan metabolism pathways, leading to 5-HT production [32]. Its production can be influenced by gut microbiota, including *Candida*, *Streptococcus*, *Escherichia*, and *Enterococcus* strains [33].

Dopamine, which is essential for reward and motivation behaviors [34], is substantially produced and stored in the intestines [26]. Gut microbiota, including *Bacillus* strains, play a significant role in its regulation [33]. Additionally, it is the precursor of norepinephrine, which modulates the behavioral response to stress and anxiety [26, 34, 35]. Examples of norepinephrine-producing bacteria are *Escherichia*, *Bacillus*, and *Saccharomyces* strains [33].

GABA is an inhibitory neurotransmitter in the central nervous system and enteric nervous system [36]. Important microbial sources of GABA are lactic acid bacteria, namely, the *Lactobacillus*, *Enterococcus*, *Leuconostoc*, *Pediococcus*, *Propionibacterium*, and *Weissella* genera [37]. GABA-ergic neurotransmission inhibits the amygdala and prevents inappropriate emotional and behavioral responses [38]. Changes in GABA regulation are linked with stress and anxiety [38], and daily GABA administration [39] and modulation of GABA-A receptor signaling [40] has been found to reduce anxiety symptoms in stressed rodents.

All these data strongly suggest the interplay between gut microbiota, neural mechanisms, and behavior; however, further studies are required to evaluate this link accurately.

#### 3.2.3. Endocrine Routes

In addition to the abovementioned pathways, gut microbiota controls endocrine mechanisms that integrate the GBA by regulating the hypothalamic-pituitary-adrenal (HPA) axis. This system is fundamental for basal homeostasis and controls several body processes in response to stress factors [41]. In stress situations, corticotropin-releasing factor (CRF) is secreted from the hypothalamus and induces adrenocorticotropic hormone (ACTH) discharge in blood circulation. In turn, glucocorticoids are secreted from the cortex of the adrenal gland. Stress events can activate the HPA axis, leading to an increase in cortisol levels [41]. The hyperactivation of the HPA axis has been associated with, anxiety traits in children with low socioeconomic environments [42], and stressful circumstances [43]. The complex feedback mechanism behind the HPA axis might be partially regulated by the gut microbiota since some bacteria might induce its overactivity, thus influencing behavior patterns [44]. On the other hand, stressful situations and further activation of the HPA axis can result in changes in gut microbiota composition [41]. Consequently, the intestinal barrier integrity might be affected, thus influencing intestinal permeability, which might induce an inflammatory response wherein mediators such as cytokines and prostaglandins can activate the HPA axis [45]. Besides the negative impact on immunologic states, a persistent hyperactivation of the HPA axis might alter the hippocampal structure, potentially resulting in altered neurogenesis, the morphology of neurons, or even cellular death [44]. In this complex interplay, a role for gut microbiota in these immune-neuroendocrine mechanisms is suggested [41].

#### 3.2.4. Immune-Mediated Mechanisms

Gut bacteria play a major role in immune system development and function. Through the modulation of the gut- and brain-resident immune cells, microbiota influence cognitive function, and behavior. Signaling pathways involved in this mechanism include the regulation of cytokines and chemokines. Furthermore, innate and adaptive immune systems influence gut microbiota composition, which is fundamental to maintain the symbiotic relationship with the host [14]. When homeostatic balance is disrupted, the gut microbiota can induce a proinflammatory state, influencing the production of cytokines and chemokines [45]. Increasing evidence suggests the role of proinflammatory cytokines in anxiety disorders [46–51]. Anxiety disorders have been associated with a specific cytokine patterns in rodents [49] and humans [45, 47, 50–52], characterized by an increased proinflammatory cytokines, such as IFN-*γ* [45, 47, 52], Tumor necrosis factor-alpha (TNF-*α)* [45, 49, 50], IL-6 [47, 50–52], IL-8 [47, 51], IL-1*α* [47], IL-2 [47], IL-1*β* [51], IL-12p70 [47], as well as reduced anti-inflammatory cytokines, including IL-10 [45]. Opposite results have been demonstrated regarding IFN-*γ* and TNF-*α*, in men exposed to high psychosocial stress [51].

In the brain, cytokines might influence the metabolism of neurotransmitters, such as 5-HT, dopamine, and glutamate; interfere with the function of the HPA axis, thus affecting the production of the involved hormones; activate the nuclear factor kappa-light-chain-enhancer of activated B cells, consequently affecting the development of neural tissues; or target brain neurocircuits that control motivation, reward, anxiety, arousal, and alarm states [47]. Hence, inflammatory cytokines can have essential roles in neurodegenerative mood disorders' onset and development stages [48].

## 4. Intestinal Permeability

The intestinal barrier is a complex functional structure that protects the organism from pathogen invasion and toxins, simultaneously allowing the absorption of nutrients and electrolyte changes [19]. Tight junctions (Tj) play an important role in this regulation as they support the barrier's integrity. These multiprotein complexes are located at the IECs, specifically at the apical ends of the lateral membranes. Tj proteins, including claudin and occludin, support its structure as they form a selectively permeable barrier in the paracellular pathways [53]. Several other elements are essential to intestinal barrier homeostasis, such as goblet cells that secrete mucin, essential to forming the outside mucus layer, which protects the epithelial surface [19]. Moreover, Paneth cells produce important antimicrobial peptides in the small intestine's crypts. Also, humoral elements, such as defensins, have a role in this regulation [19].

Inflammatory states emerging from diseases might induce metabolic, immunologic, and neuroendocrine alterations that influence the integrity of this barrier, affecting gut permeability [19, 54]. On the other hand, dysbiosis may lead to the loss of intestinal barrier integrity, resulting in the movement of luminal content, namely, pathogens, toxins, and antigens, to the bloodstream [19], which might induce an imbalanced mucosal immune system as well as an inflammatory state, with the production of proinflammatory cytokines [53, 55]. Alterations in intestinal permeability can either precede or appear secondary to an inflammatory status involved in diseases [15]. Chronic stress situations and sleep deprivation can lead to alterations in microbiota composition, suggesting the interplay between both factors [56].

## 5. Prebiotics and Probiotics

Considering the mechanisms involved in regulating the GBA, microbiome modulation stands out as a promising therapeutic solution for anxiety disorders [16].

According to the International Scientific Association for Probiotics and Prebiotics consensus statement (ISAPP), the term probiotic was revised to “live microorganisms that, when administered in adequate amounts, confer a health benefit on the host” [57]. Probiotics can impact the regulation of the central and enteric nervous systems through different mechanisms that include attenuation of the HPA axis, restraining cytokine production, and influence the neuroendocrine system. The use of probiotics, such as *L. plantarum* P-8 [44], various *Streptococcus*, *Bifidobacterium*, *Lactobacillus*, and *Lactococcus* strains [48, 58], and *Lactobacillus rhamnosus* HN001 [59], has been shown to improve stress, anxiety [16, 44, 48, 58], memory, and cognitive potential in humans [16, 44, 48, 59]. Additionally, the use of probiotics induced beneficial changes in gut microbiota, increasing diversity and influencing specific bacterial species [16]. Alongside, probiotic supplementation also led to reduced proinflammatory cytokines such as IFN-*γ* and TNF-*α* [44], and potentially lowered plasma cortisol [44]. Probiotics might also influence metabolite production, such as SCFAs, and neurotransmitters, including 5-HT and GABA. This strongly suggests their multidimensional action in the GBA [60].

More recently, the ISAPP has also updated the definition of prebiotics to “a substrate that is selectively utilized by host microorganisms conferring a health benefit” [61]. These nondigestible compounds act as nutrient sources, promoting the growth of beneficial bacteria (probiotics) in the colon [60]. Prebiotics more widely accepted for their clinical evidence are fructooligosaccharides (FOS) and galactooligosaccharides (GOS). Others include mannan oligosaccharides (MOS), xylooligosaccharides (XOS), human milk oligosaccharides, inulin, conjugated linoleic acid, polyunsaturated fatty acids, phenolics, and phytochemicals [62]. Prebiotic administration, including lactoferrin [63], FOS [63–65], GOS [63, 64, 66, 67], and polydextrose [63], has been found to improve anxiety symptoms in both humans [65–67] and rodents [63, 64]. It has also resulted in changes in the gut microbiota, specifically increases in *Lactobacillus* spp. [63] and *Bifidobacteria* [65]. Counterintuitively, some results have shown a decrease in *Bifidobacterium* and *Lactobacillus* spp., which could be explained by the enhancement of indigenous commensals through prebiotic administration, reducing the relative abundance of these added probiotics [64]. Prebiotic supplementation also showed a reduction in proinflammatory cytokines, such as TNF-*α* [64], increased acetate and propionate, but decreased isobutyrate levels [64]. Additionally, it appeared to reduce hyperactivation of the HPA [64, 67] and lower tryptophan levels, possibly due to increased bacterial uptake or the production of 5-HT [64]. Additionally, the combined administration of *Lactobacillus casei* and inulin significantly improved anxiety symptoms in rodents more effectively than either treatment alone, impacting their endocrine and neurochemical systems [62]. The term “psychobiotic” has been attributed to probiotics that offer health benefits to individuals with psychiatric conditions. These microorganisms produce neuroactive substances that influence the GBA and hold potential in alleviating symptoms of disorders such as depression and anxiety [68]. This definition also encompasses prebiotics, which contribute to the proliferation of beneficial gut bacteria [69].

This novel concept is well known for its anti-inflammatory, antidepressant, and antianxiety effects [60]. The strength of these formulations is that they lack cognitive side effects, nor are they addictive, like other drugs usually prescribed to treat anxiety disorders [70]. Thus, psychobiotics appear as a novel alternative for the treatment of anxiety disturbances [16, 60, 70].

## 6. Recent Advances in the Veterinary Behavioral Medicine

In veterinary practice, there have been remarkable advances in neurodevelopment research associated with the canine gut microbiome.

Canine aging and cognitive performance have been associated with dysbiosis. In a study involving 29 mixed breed dogs, behavioral tests were conducted to assess cognitive performance. The results showed a lower proportion of *Fusobacteria* in older dogs and fewer *Actinobacteria* in dogs with higher memory faculties [71]. Canine aggressiveness, a type of anxiety-like behavior, was evaluated in a group of 31 American Pit Bull Terriers [72]. Whereas, aggressive dogs showed a higher proportion of *Firmicutes*, particularly members of the *Lactobacillus* genus; dogs lacking this behavior demonstrated a higher abundance of Proteobacteria and *Fusobacteria* strains. It is relevant to highlight that dogs exhibiting non-aggressive behavior showed a higher abundance of *Bacteroides* and *Dorea* strains from the *Bacteroidetes* and *Firmicutes* phyla, respectively, compared to aggressive dogs. These results led to the following question: are the detected alterations in microbiota composition a predisposing factor or a consequence of canine aggressiveness? A third option also considered the possibility of unexplored variants intrinsic to dogs with aggressive behavior that could influence their microbiota [72]. A preliminary study established a correlation between behavior phenotypes and specific gut microbiota structures in a sample of 42 dogs of various breeds [73]. The authors suggested that a chronic stress scenario might influence the gut's internal environment by interacting with neuroactive metabolites secreted by commensal bacteria, thereby influencing host behavior. A behavioral specialist grouped dogs as aggressive, phobic, and healthy, and those with aggressive behavior had a lower abundance of *Bacteroidetes*. This group of dogs exhibited a lower abundance of members of the *Oscillospira*, *Peptostreptococcus*, *Bacteroides*, *Sutterella*, and *Coprobacillus* genera. Furthermore, an increase of typically low-prevalence bacteria was detected in aggressive dogs, including *Dorea*, *Blautia*, *Collinsella*, and *Slackia*, with an even higher prevalence of strains of *Catenicabacterium* and *Megamonas* [73]. Concerning dogs with phobic disorders, no substantial alterations were detected at the phylum level. Surprisingly, canine phobic behavioral disorders were associated with a higher proportion of *Lactobacillus*, a genus usually found predominantly in probiotic formulations. The authors also analyzed fecal cortisol and testosterone levels, showing no significant correlation between these and aggressive or phobic disorders. Although the population cohort in this study was not sufficient to draw significant conclusions, this investigation undoubtedly paved the way for further large-scale studies [73].

Regarding neuroendocrine mechanisms, a recent study investigated the relationship between 5-HT and tryptophan and dogs' behavioral response to stress stimuli [74]. A mixed-breed group of 39 healthy shelter dogs was classified according to their behavioral response to medical examination and blood collection procedures. Serum 5-HT and tryptophan concentrations were analyzed, and no correlation was established. This could be because none of the dogs in this study displayed intense behavioral disorders. Another possible explanation suggested by the authors is the opposite way of circulation of 5-HT and tryptophan in the BBB, which regulates the passage between central and peripheral circulation [74]. Likewise, a 107 mixed-breed group of dogs with different levels of aggressive, fearful, and impulsive behaviors, defined accordingly to the owner-answered questionnaires, did not show significant alterations in serum 5-HT and cortisol levels after being restrained when exposed to a novel environment [75]. It is possible that the exposition to a new environment could not be sufficiently distressful to cause alterations in these markers' concentrations or that the methods of behavioral evaluation performed were not accurate enough. Another possible explanation was the lack of a relationship between these markers and canine behavioral tendencies [75].

Regarding probiotic therapeutic use, a fourteen-day supplementation of *Lactiplantibacillus plantarum* PS128 seemed to stabilize aggression and separation anxiety behaviors in dogs. Moreover, plasma 5-HT turnover ratio decreased after supplementation, specifically in dogs with separation anxiety. This implicates 5-HT as a potential factor in the GBA, indicating a slower breakdown of 5-HT into its metabolites, and consequently a higher availability of this neurotransmitter in the system [76].

Furthermore, Purina® researchers have highlighted the potential benefits of *Bifidobacterium longum* (BL999) in dogs with anxiety behaviors in a study supporting a new product (https://www.purinaproplanvets.com/media/521317/086602_vet1900–0918cc_abstract.pdf). Involving 24 anxious Labrador Retrievers, the study assessed the effects of BL999 on behavior and physiological markers like heart rate and salivary cortisol. Over a 12-week period, with a midway washout, dogs alternated between BL999 and a placebo. Significant improvements were noted in anxious behaviors and physiological markers in the BL999 group. These preliminary findings are promising, and releasing comprehensive study details for peer review would greatly benefit the scientific community, enabling further evaluation and expansion upon these results. A double-blind, placebo-controlled clinical trial probed the effects of a novel nutraceutical supplement in canine stress-related behaviors and its relationship with the fecal microbiome [77]. Relaxigen Pet dog® contains a mixture of prebiotics (FOS), probiotics (*Lactobacillus reuteri*), post-biotics (butyric acid), 5–hydroxytryptophan (5–HTP), a 5-TH precursor, L-theanine, derived from glutamine, a precursor of GABA, and other natural inflammatory compounds, including conjugated linoleic acid, a neuroprotective supplement with anti-inflammatory properties, and Krill, an Omega-3 rich oil. Forty dogs enrolled in this study were classified by a veterinary behaviorist according to their stress behavior level. Anxious dogs treated with Relaxigen Pet dog® had lower levels of *Bacteroides*, *Prevotella*, *Porphyromonas*, *Bifidobacterium*, *Lactobacillus*, and *Enterobacteriaceae* strains. This group also demonstrated less anxiety-like behaviors [77]. This innovative study showed promising results, which will bring new insights into veterinary behavioral medicine and pave the way for future research. To our knowledge, no other research has been conducted on the utilization of probiotics for the treatment of canine anxiety disorders.

Despite recent significant progress in veterinary behavioral medicine, the link between canine anxiety disorders and the gut microbiome, as well as the potential benefits of probiotics in this condition, remains to be further elucidated and tested on a larger scale.

## 7. Conclusions

Canine anxiety disorders present a significant issue in veterinary behavior today, posing medical challenges and affecting both pet and owner quality of life.

The influence of microbiota on mental and behavioral disorders, including anxiety, is well-documented in human and rodent models, considering the metabolic, neuroendocrine and immune-mediated mechanisms involved. However, there is a significant gap in understanding these relationships in the context of canine veterinary behavior.

In this study, several conclusions have been drawn:

1. The limited number of studies included on canine anxiety disorders and GBA reflects the scarcity of research on this topic. Current studies are broad and focus on a few specific markers only. In addition, most clinical trials relied solely on behavioral patterns to assess anxiety. Future studies should incorporate more biomarkers such as specific cytokines, metabolites, hormones, neurotransmitters, and others to accurately evaluate canine anxiety levels.
2. Extensive research in human and rodent models has elucidated the role of microbiota in behavioral disorders. The authors propose that leveraging this knowledge could be pivotal in advancing canine research and enhancing veterinary practices.
3. Despite microbiota differences among humans, rodents, and dogs, one further step in the investigation on this topic could be the assessment of specific microbiota in dogs, which have been previously associated with anxiety symptoms in humans and rodents.
4. Regarding prebiotic and probiotic therapies, to our knowledge, there have only been two peer-reviewed studies conducted on dogs [76, 77]. Although promising, it is not enough to draw firm conclusions, indicating the need for further research.

Continuous efforts in this area are likely to shed light on the impact of GBA on canine anxiety disorders, as well as on its treatment, potentially improving both dog's and owner's quality of life. Additionally, this would represent a significant milestone in the field of veterinary behavioral medicine.

## Figures and Tables

**Table 1:** List of the behaviors evaluated in the Lincoln Canine Anxiety Scale (adapted from [3]).

Behaviors	Scores		
	0	1	5
Running around		Occasional burst of activity	Continuously running around
Drooling saliva		Damp around mouth	Pools of saliva
Hiding (e.g., under furniture and behind owner)		Retreats	Will not be removed from hiding area
Destructiveness (e.g., furniture, carpets,…)		Small items, e.g., pens	Extensive amount, e.g., holes in the wall
Cowering (e.g., tucks tail and flattens ears)		Uneasy	Petrified
Restlessness/pacing		Small amount	Extensive amount fixed route continuously traced
Aggression (e.g., growling, snapping, or biting)		Occasional growl	Severe biting attempts made
“Freezing to the spot”		Occurs sporadically within an event	Most of the time
Barking/whining/howling		Small amount	Extensive amount
Panting		Occurs sporadically within an event	Most of the time
Vomiting/defecating/urinating/diarrhea	Not present	Small amount	Extensive amount
Owner-seeking behavior		Seeks out owner occasionally during the event	Will not leave owner in any circumstance
Vigilance/scanning of the environment		Occurs sporadically within an event	Most of the time
Bolts		Occurs occasionally in response to certain noises	Occurs always in response to a wide range of sounds
Self-harm		Small amount, e.g., licking feet	Extensive amount e.g., broken teeth or nails
Shaking/trembling		Occurs occasionally in response to certain noises	Occurs always in response to a wide range of sounds

## Abbreviations

ACTH: Adrenocorticotropic hormone
BBB: Blood-brain barrier
CRF: Corticotropin-releasing factor
EC: Enterochromaffin cells
FOS: Fructooligosaccharides
GABA: Gamma-aminobutyric acid
GBA: Gut-brain axis
GI: Gastrointestinal
GOS: Galactooligosaccharides
HPA: Hypothalamic-pituitary-adrenal
IFN-*γ*: Interferon-gamma
IL: Interleukin
ISAPP: International Scientific Association for Probiotics and Prebiotics consensus statement
MOS: Mannanoligosaccharide
SCFAs: Short-chain fatty acids
Tj: Tight junctions
TNF-*α*: Tumor necrosis factor-alpha
XOS: Xylooligosaccharide
5-HT: Serotonin
5-HTP: 5-hydroxytryptophan.
